# Network Pharmacology and Molecular Docking Analysis Explores the Mechanisms of *Cordyceps* s*inensis* in the Treatment of Oral Lichen Planus

**DOI:** 10.1155/2022/3156785

**Published:** 2022-08-29

**Authors:** Hexin Ma, Guofang Wang, Xiaomeng Guo, Yao Yao, Chunshen Li, Xibo Li, Mingzhe Xin, Xiaohui Xu, Shilong Zhang, Zhi Sun, Hongyu Zhao

**Affiliations:** ^1^Department of Oral Emergency, The First Affiliated Hospital of Zhengzhou University Stomatological Hospital of Henan Province, Zhengzhou 450000, China; ^2^School and Hospital of Stomatology of Zhengzhou University, Zhengzhou 450000, China; ^3^Department of Oral Medicine, The First Affiliated Hospital of Zhengzhou University Stomatological Hospital of Henan Province, Zhengzhou 450000, China; ^4^Guangzhou Institutes of Biomedicine and Health, Chinese Academy of Sciences, Guangzhou 510530, China; ^5^Department of Pharmacy, The First Affiliated Hospital of Zhengzhou University, Zhengzhou 450000, China; ^6^Henan Engineering Research Center of Clinical Mass Spectrometry for Precision Medicine, Zhengzhou 450000, China

## Abstract

**Objective:**

Oral lichen planus (OLP) is the most common potentially malignant disorder of the oral cavity. This study aimed to investigate the mechanism of action of *Cordyceps sinensis* in the treatment of OLP and provides a theoretical support for improving current treatment regimens for OLP.

**Methods:**

The active components and therapeutic targets of *Cordyceps sinensis* were predicted and screened using the TCMSP, SymMap, PubMed, HIT 2.0, and PharmMapper databases, while the relevant OLP targets were predicted and screened using the DisGeNET and GeneCards databases. Protein-protein interactions (PPI) were examined using the String database, and Cytoscape was used to combine and illustrate the findings. GO and KEGG pathway enrichment analyses were carried out using RStudio, and AutoDock Vina and Pymol were used for molecular docking and visualization, respectively.

**Results:**

A total of 404 potential target genes were discovered after evaluating 21 active compounds from *Cordyceps sinensis*. Potential therapeutic targets included 67 targets that matched and overlapped with OLP, including TNF, IL-6, CD4, EGFR, and IL1B. Key targets were predominantly engaged in the PI3K-Akt signaling pathway and the MAPK signaling pathway, according to the GO and KEGG analyses. These targets have a connection to biological processes including apoptosis signaling pathway regulation, T cell activation, and oxidative stress response. The molecular docking results showed that TNF, IL-6, CD4, EGFR, and IL1B could bind to their corresponding active components.

**Conclusions:**

*Cordyceps sinensis* contains multiple components and acts on multiple targets and multiple pathways. Particularly, *Cordyceps sinensis* targets TNF, IL-6, CD4, EGFR, and IL1B, regulates PI3K-Akt and MAPK signaling pathways, as well as takes part in biological processes including apoptosis, T cell activation, and oxidative stress. *Cordyceps sinensis* could be a crucial choice in the therapy of OLP.

## 1. Introduction

Oral lichen planus (OLP) is a chronic or recurrent inflammatory autoimmune disease of the oral mucosa, with an incidence of 1% worldwide and substantial regional variation [1]. The WHO has identified OLP as an oral potentially malignant condition (OPMD) due to evidence that it has malignant potential. Its most dangerous consequence is the development of oral squamous cell carcinoma (OSCC) [2]. Currently, there is no cure for this disease. Adrenocorticosteroids and immunosuppressants are commonly used to reduce inflammation and promote healing. Although certain efficacy has been achieved, the disease is prone to recurrence, and long-term hormone therapy has significant side effects, such as secondary candidiasis, mucosal atrophy, and dryness [3, 4]. Thus, it is crucial to choose medications that may properly cure the condition without causing major adverse reactions.

In recent years, Chinese herbal medicine has achieved results in the treatment of OLP, such as Liuwei Dihuang [5], Tripterygium glycosides [5], curcumin [6], and aloe vera [7]. Their mechanisms of action may be multifaceted, such as correcting the imbalance of T-lymphocyte subsets, inhibiting inflammatory responses, antioxidative stress, and increasing cytokine release from macrophages. However, there is still an urgent need for innovative drugs for the treatment of OLP.

With a multifactorial etiology and malignant transformation tendency, OLP has been substantially studied; however, its pathophysiology and etiology remain elusive [8]. It is believed that immune dysregulation plays a crucial role in the development of OLP and the primary lymphocytes implicated are believed to be CD8+ cytotoxic and CD4+ Th1 polarized T cells, which are driven by the identification of nonself antigens, activating T cell subsets that are directed towards oral keratinocytes and causing apoptosis of keratinocytes [9]. The abnormalities of cytokines, like IL-1, TGF-*β*, IFN-*γ*, TNF-*α*, and others, produced during the development of OLP can lead to immunodeficiency, allergy, and autoimmunity [10]. An imbalance in redox homeostasis in OLP was shown by a recent meta-analysis that revealed an increase in oxidative stress markers and a significant decline in antioxidant markers in OLP patients compared to healthy controls [11]. The diversity of etiology and pathogenesis makes its treatment lack a clear or uniform model. Therefore, the characteristics of Chinese medicine, with multicomponent and multitarget, may offer a fresh perspective for the treatment of OLP.

*Cordyceps sinensis*, a traditional Chinese medicine, is mainly distributed in alpine zones with a wide variety of species. The majority of them are entomopathogenic fungus that infects insect larvae and pupae [12]. Research has found that *Cordyceps sinensis* includes a wide variety of chemical elements, including nucleosides, sugars, sterols, proteins, and sphingolipids [13]. Research has shown that *Cordyceps sinensis* and its bioactive molecules have a variety of pharmacological effects, including anti-inflammatory, antioxidant, antitumor, antihyperglycemic, antiapoptotic, immunomodulatory, nephroprotective, and hepatoprotective properties [14]. Based on several pattern recognition receptors (PRR), cordycepin polysaccharide has an in vitro immunostimulatory activity by activating mitogen-activated protein kinase (MAPK) and nuclear factor-*κ*B (NF-*κ*B) signaling pathways, inducing the production of nitric oxide (NO), reactive oxygen species (ROS), and more [15]. By increasing splenocyte proliferation, natural killer (NK) cell activity, levels of cytokine, as well as reducing glutamate-induced oxidative stress and oxidative stress-related apoptosis, cordycepin successfully fights against the immunosuppressive effects of cyclophosphamide [16, 17]. Without altering human fibroblasts, cordycepin inhibits epithelial-mesenchymal transition (EMT) and induces apoptosis to prevent OSCC [18]. More and more data points to the possibility that *Cordyceps sinensis* and its preparations might cure OLP via antioxidant, immunomodulatory, and anti-inflammatory mechanisms. In China, *Cordyceps sinensis* preparations (e.g., Bai Ling capsule and Jin Shui Bao capsule) are clinically effective as adjuvant therapeutic agents for OLP, but the specific mechanisms are not yet clear.

Network pharmacology is an approach to predict disease-specific targets from biomedical data available in systems biology and polypharmacology [19]. With the development of biomedical data, new methods based on “active ingredient-target-disease” and interaction networks have been developed to understand the complex pharmacological mechanisms of Chinese medicine, and have blossomed in recent years [20, 21]. Network pharmacology's fundamental principles and the holistic view of Chinese medicine are intertwined, leading to a change from the traditional “one target, one drug” paradigm to the more recent “multicomponent, multitarget” model, which is the most effective model for addressing multitarget medications [22, 23]. In recent years, several studies have successfully elucidated the drug-component-target-proteins and their mechanisms of action on diseases through network pharmacology approach [24–26]. However, there is no relevant report on the connection between *Cordyceps sinensis* and OLP.

**Figure 1:**
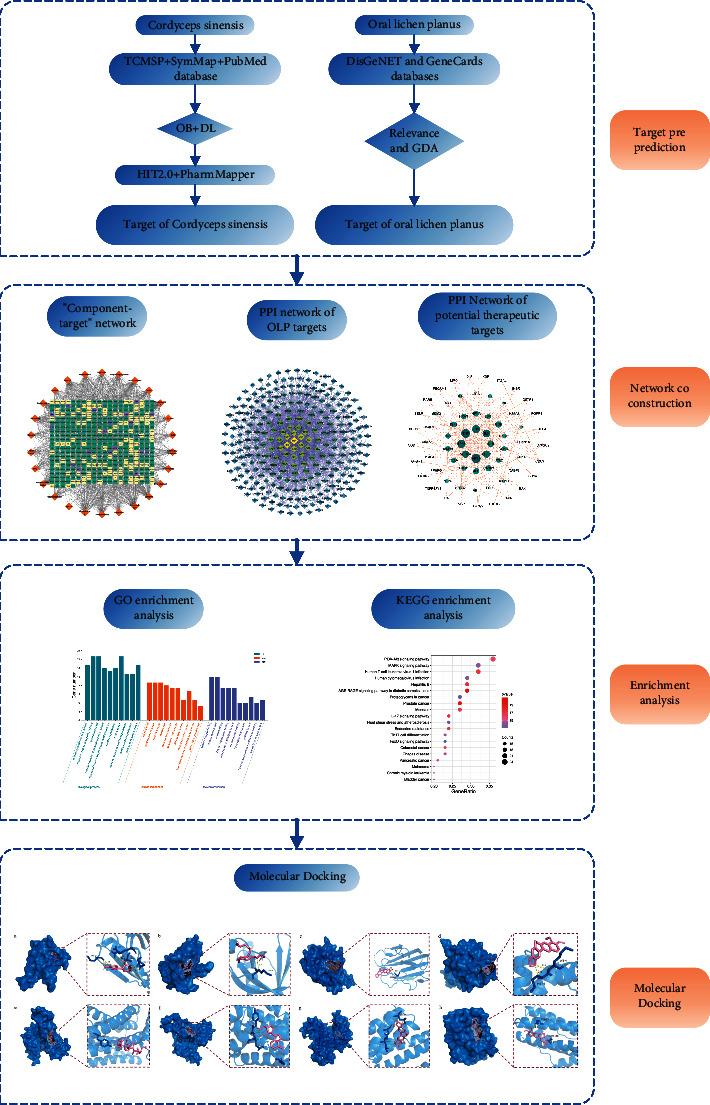
Diagram of the research workflow.

In order to examine the possible targets of the active components of *Cordyceps sinensis* for the treatment of OLP, we conducted this study using a network pharmacology and molecular docking approach. This study will provide a framework for further research into the pharmacological processes by which *Cordyceps sinensis* works to cure OLP. The procedure of this study is displayed in Figure 1.

## 2. Materials and Methods

### 2.1. Identiﬁcation of Active Components

The compounds of *Cordyceps sinensis* were searched in the TCMSP [27] (https://tcmspw.com/tcmsp.php) and SymMap [28] (https://www.symmap.org/) databases with *Cordyceps sinensis* as the keyword, and oral bioavailability (OB) ≥ 30% and drug-like quality (DL) ≥ 0.18 (a screening threshold of TCMSP database) [27]. In addition, the PubMed database search (https://pubmed.ncbi.nlm.nih.gov) was added to include active ingredients that did not meet the above criteria but had significant pharmacodynamic effects or high levels. Finally, the active compounds of *Cordyceps sinensis* were identified and the active ingredient structures were acquired using the MOL2 format files on the TCMSP platform.

### 2.2. Target Collection and Prediction of *Cordyceps sinensis*

HIT2.0 (Herbal Ingredients' Targets Platform) (https://www.badd-cao.net:2345/) is an evidence-based comprehensive search and management platform for herbal ingredients and target information [29], where active ingredients are entered to obtain known targets. In addition, PharmMapper is used to identify potential candidate targets for a given small molecule drug by using a pharmacophore mapping approach [30] by submitting the MOL2 format file of the active ingredient to the PharmMapper platform(https://www.lilab-ecust.cn/pharmmapper/) for target prediction. Human Protein Targets Only is selected on the “Select Targets Set,” and the rest of the parameters are kept as default settings. Results obtained from the PharmMapper platform for each active ingredient are used for subsequent analysis based on the targets selected with *Z*′-score > 1. Finally, all of the target proteins identified during the screening are annotated into gene names using the UniProt database (https://www.uniprot.org/), excluding any nonhuman targets.

### 2.3. Selection of OLP Targets

The OLP-related targets were retrieved from the DisGeNET (https://www.disgenet.org/) and GeneCards databases (https://www.genecards.org) using “oral lichen planus” as the keyword. To increase the reliability of the findings, the targets obtained from the GeneCards and DisGeNET databases were filtered for results that had a relevance score ≥ 5 and GDA ≥ 0.01, respectively.

### 2.4. The Possible OLP Therapy Targets of *Cordyceps sinensis* and Network Construction

To create a Venn diagram and determine the common targets of *Cordyceps sinensis* and OLP treatment, the targets of the active substances of *Cordyceps sinensis* and OLP were entered into the R software using the Venn diagram. Then, the targets of the ingredients and diseases were loaded into Cytoscape 3.9.0 (https://www.cytoscape.org/), and a “component-target” network was created using the Merge function.

### 2.5. PPI Network Construction

The STRING database (https://www.string-db.org/) was used to create the protein-protein interaction network (PPI). The potential targets of *Cordyceps sinensis* for OLP treatment in the “multiple proteins” section were entered and the species was set as “*Homo sapiens*.” The “high confidence (0.700)” was selected as the confidence level to obtain the PPI network related to the effectiveness of *Cordyceps sinensis*. The network was then loaded into Cytoscape for visualization, and the topological parameters of the nodes in the network were calculated using the app CytoHubba.

### 2.6. Functional Enrichment Analysis

Gene Ontology (GO) and Kyoto Encyclopedia of Genes and Genomes (KEGG) pathway enrichment analyses were performed using the ClusterProﬁler package of R programming language, and a screening criterion of *p* adjust < 0.05 and *q* value < 0.2 was used. Then, the significant enrichment results were plotted by the “ggplot2” package. Heatmap was created using the online data analysis and visualization tool (https://www.bioinformatics.com.cn/).

### 2.7. Molecular Docking

The five core proteins with the greatest node degree values in the PPI network were docked to their active components. The RCSB PDB database (https://www.rcsb.org/) was used to obtain the protein crystal structures, while the TCMSP database was used to download the compound MOL2 structures. They were processed using AutoDock Tools (https://mgltools.scripps.edu/documentation/links/autodock), including separation of the protein structure from the original ligand, removal of water molecules and charge added to the structure, and converted to “PDBQT” format. The 3D docking photos were created using Pymol 2.4.0 (https://pymol.org/2/) software. According to research studies, when the binding energy of a small molecule medication to a protein is less than −4.25 kcal/mol, it is considered to have binding activity between the two. Additionally, two molecules have excellent binding activity when the binding energy is less than −5.0 kcal/mol [31].

## 3. Results

### 3.1. Active Compounds of *Cordyceps sinensis*

The numbers of drug ingredients retrieved from the TCMSP and SymMap databases were 38 and 153, respectively, with a total of 153 after deduplication. Twenty-one active substances were tested using the standards and five more items that did not pass the screening standards were included based on the literature [13, 32–34], making a total of 26 active ingredients (Table 1).

### 3.2. The Construction of the “Component-Target” Network

**Figure 2:**
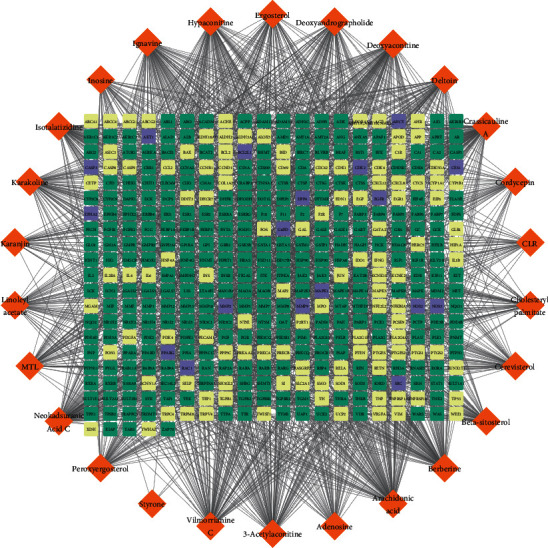
Network of “component-target” in *Cordyceps sinensis*. The target is represented by the rectangle, while the component is symbolized by the orange diamond. The expected targets are represented by green rectangles, while the known targets are represented by yellow rectangles, and purple represents targets that are common to both predicted and known.

The 26 active components in *Cordyceps sinensis* were searched in the PharmMapper and HIT 2.0 databases, yielding 293 and 130 targets, respectively, and 404 after deduplication (Supplementary Table 8). The *Cordyceps sinensis*“ingredient-target” network was then created using Cytoscape 3.9.0 (Figure 2). As seen in Figure 2, there were 274 anticipated targets, which made up 67.8% of the total targets, and 112 targets based on literature evidence, which made up 27.7% of the total targets. Between the projected targets and the objectives supported by the literature, there were 18 common targets. Meanwhile, network analysis revealed that arachidonic acid, berberine, neokadsuranic acid C, deoxyaconitine, and vilmorrianine C were the main active ingredients for treating OLP, and these components regulated 116, 116, 107, 105, and 103 targets, respectively.

### 3.3. PPI Network of OLP Targets

**Figure 3:**
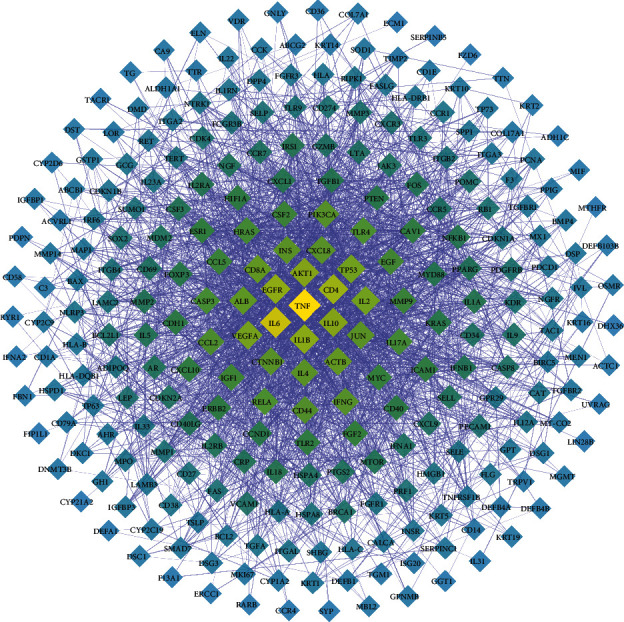
PPI network of OLP targets, the diamond represents the target, the colors range from yellow to blue, the sizes range from large to tiny, and the transparency range from low to high represents the Degree values ranging from large to small.

A total of 293 targets were identified after de-duplication from the search of OLP-related genes in the GeneCard and DisGeNET databases (Supplementary Table 8). A PPI network (Figure 3) was established to show the association between targets connected to OLP, and 52 targets were found to be greater than the average of degree centrality (DC), closeness centrality (CC), and betweenness centrality (BC) at the same time (Supplementary Table 8). Among these targets, TNF, IL-6, CD4, EGFR, IL1B, IL10, AKT1, VEGFA, TP53, and IL2 had the highest degree values, indicating that these targets are important in the development of OLP and are expected to be targeted for clinical treatment of OLP.

### 3.4. PPI Network of Treatment Targets

**Figure 4:**
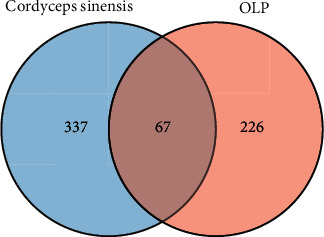
The Venn diagram of *Cordyceps sinensis* and OLP targets.

**Figure 5:**
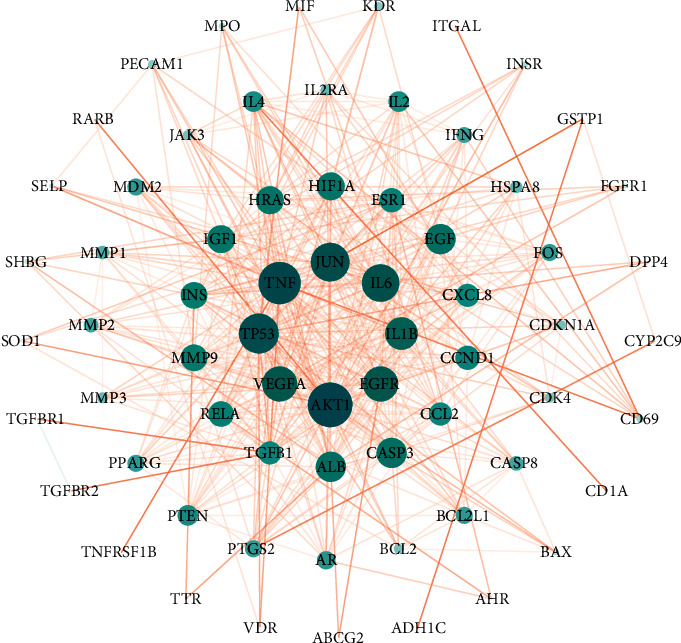
PPI network for conceivable therapeutic targets. Proteins are shown by the circles, while the connections between proteins are shown by the lines. The node degree values are shown as tiny to big circles and bright to dark hues. Lines that go from thin to thick show the transition from tiny to enormous.

The intersection of the target genes of *Cordyceps sinensis* and OLP was obtained by using the Venn diagram R software package, and the 67 common targets (Supplementary Table 8) were identified to be the prospective targets of *Cordyceps sinensis* in the treatment of OLP (Figure 4). The protein interaction network of potential therapeutic targets is shown in Figure 5, where 468 interactions exist for 67 targets in the graph. We filtered 32, 34, and 21 important nodes in the network by three parameters, including degree centrality (DC), closeness centrality (CC), and betweenness centrality (BC), respectively. In addition, the DC, CC, and BC of 19 nodes, including AKT1, TNF, and TP53, exceeded the average values of the topological parameters of the whole network, demonstrating that these 19 targets might be the main targets of *Cordyceps sinensis* in the treatment of OLP (Table 2).

### 3.5. Functional Analysis

#### 3.5.1. GO Enrichment Analysis Results

**Figure 6:**
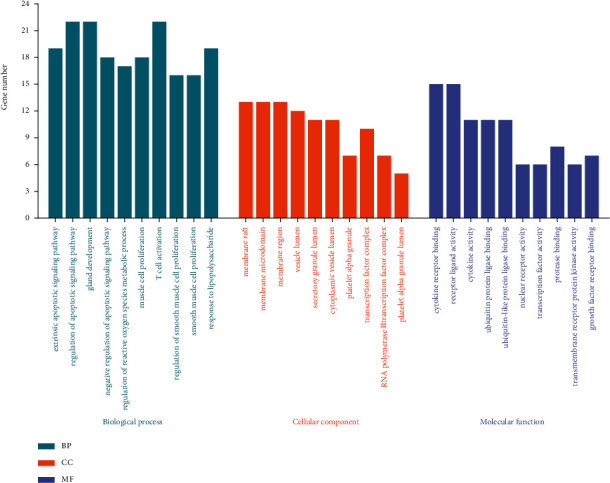
GO enrichment outcomes. The top 10 GO items in each category are shown by the horizontal axis, while the number of genes enriched in each entry is represented by the vertical axis.

We obtained a total of 2234 GO entries for possible treatment targets of *Cordyceps sinensis*, including 2100 entries for biological processes (BP), 34 entries for cellular components (CC), and 100 entries for molecular function (MF). The top 10 GO entries (Supplementary Table 8) enrichment results are shown in Figure 6. Regarding BP, the potential therapeutic targets are chiefly focused on extrinsic apoptotic signaling pathway, muscle cell proliferation, regulation of apoptotic signaling pathway, etc. For CC, the main targets are mainly involved in membrane raft, membrane microdomain, membrane region, and others, whereas entries for MF are primarily focalized on cytokine receptor binding, receptor-ligand activity, cytokine activity, and others.

#### 3.5.2. KEGG Enrichment Analysis Results

**Figure 7:**
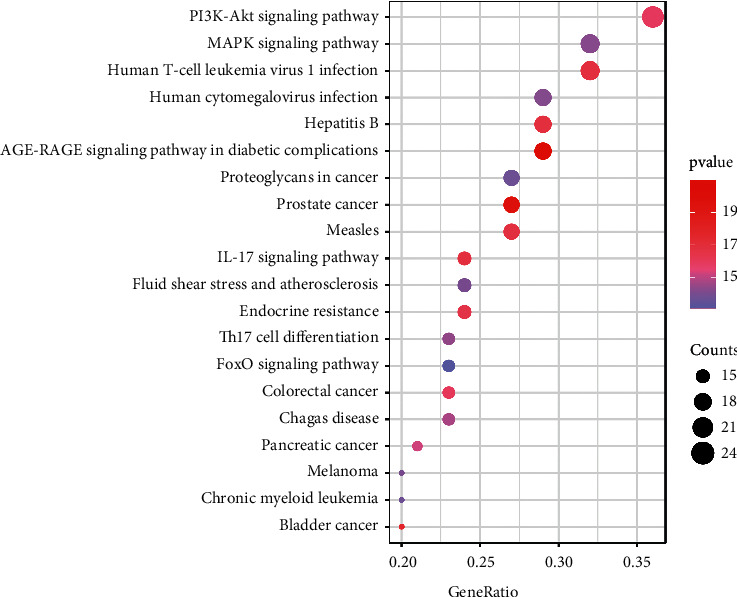
KEGG pathway enrichment of 67 putative medicinal targets. The size of the circle shows the number of genes, and the color from purple to red represents the decreasing *p* value. The horizontal axis represents the ratio of enriched genes to the total number of genes; the vertical axis represents the top 20 pathways chosen using the *p* < 0.05 criterion.

A total of 142 KEGG pathways with significant enrichment of *Cordyceps sinensis* potential therapeutic targets were obtained under the conditions of *p* adjust < 0.05 & *q* value < 0.2. The bubble diagrams were created by ranking the top 20 signaling pathways (Supplementary Table 8) according to how many enriched targets they each had in ascending order (Figure 7), among which the PI3K-Akt signaling pathway, MAPK signaling pathway, and Human T-cell leukemia virus 1 infection ranked the top three.

### 3.6. Molecular Docking

**Figure 8:**
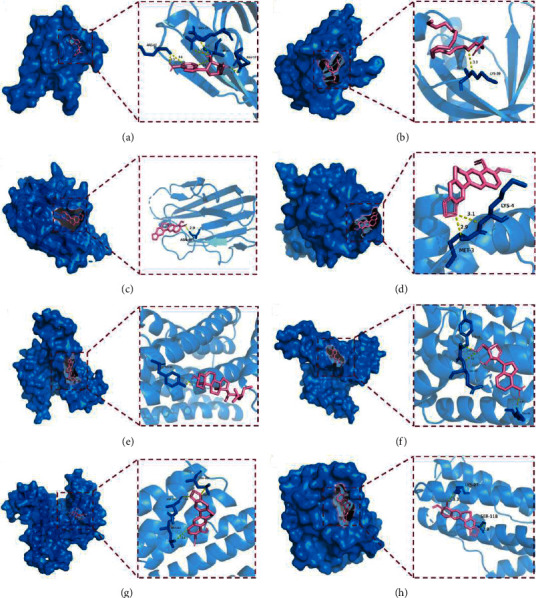
Molecular docking of the five core targets with their active ingredients. (a) The binding mode of AKT1 complexed with adenosine. (b) The binding mode of AKT1 complexed with arachidonic acid. (c) The binding mode of TNF complexed with berberine. (d) The binding mode of TP53 complexed with berberine. (e) The binding mode of JUN complexed with beta-sitosterol. (f) The binding mode of JUN complexed with cordycepin. (g) The binding mode of JUN complexed with berberine. (h) The binding mode of IL-6 complexed with berberine.

The five key targets in the PPI network, AKT1, TNF, TP53, JUN, and IL-6, were selected to obtain PDB ID and target structures, and the five targets were docked with their corresponding active ingredients (Table 3). The average binding energy for molecular docking is −5.76 kcal/mol, which is lower than −5 kcal/mol indicating that the target proteins and compounds have strong binding. The docking pattern analysis is shown in Figure 8. As shown in the figure, all active components of *Cordyceps sinensis* penetrate deeply into the active site and form polar or nonpolar bonds with key amino acid residues inside the active area to stabilize the binding of ligands and receptors. As shown in Figure 8 a, six hydrogen bonds were formed between adenosine and the active site of AKT1 involving residues ARG-67, ARG-15, THR-87, and GLU-17.

## 4. Discussion

Hitherto, the pathophysiology of OLP is still not fully understood. However, studies indicate that immunological and psychological variables may be involved [9, 35]. In recent years, despite the good efficacy of OLP treatment, the recurrent rate of OLP is still high and the side effects of long-term hormonal treatment cause a great disturbance to patients' quality of life [36]. Some studies have highlighted the effectiveness of using TCM to delay the progress of OLP and strengthen the therapeutic theory of TCM for OLP treatment [6, 7]. China has been using *Cordyceps sinensis* as medicine for more than 300 years, and modern medicine has confirmed the wide range of pharmacological effects of *Cordyceps sinensis* and its preparations, which are mainly used to treat sexual dysfunction, postillness weakness, chronic kidney disease (CKD), inflammation, and cancer [37].

For the first time, network pharmacology has been used to uncover the mechanism of action of *Cordyceps sinensis* on OLP and to give pertinent data for additional preclinical or clinical investigations. Through database search and screening, 21 active ingredients and 67 shared targets of *Cordyceps sinensis* were identified. Through PPI analysis, the main targets of *Cordyceps sinensis* for OLP treatment, including AKT1 (Degree = 38), TNF (Degree = 36), TP53 (Degree = 34), JUN (Degree = 33), and IL-6 (Degree = 32) were identified. These targets were suggested to have a significant impact on the improvement of OLP using *Cordyceps sinensis* treatment.

AKT1 is one of the three serine/threonine protein kinases (AKT1, AKT2, and AKT3) that are known as AKT kinases. These kinases control a variety of functions, such as angiogenesis, cell survival, growth, proliferation, and metabolism [38, 39]. Zhang et al. discovered that both local T cells and OLP lesions had considerably higher levels of p-Akt and p-mTOR expression, suggesting that activated Akt/mTOR autophagy may be involved in the local T-cell-mediated immune regulatory mechanisms of OLP [40]. According to several studies, Akt/mTOR activation occurs in OLP and may increase the risk of developing cancer [41, 42]. Adenosine has been shown to promote apoptosis in head and neck squamous cell carcinoma through the PI3K/Akt/mTOR signaling pathway [43]. Numerous investigations have demonstrated that the blood, saliva, or MSCs of OLP patients express more TNF and IL-6 than healthy controls [44–46], which might be an important factor in the immunopathogenesis of OLP and show an immune deregulatory condition [10]. Additionally, the probability of OLP susceptibility was substantially correlated with the inheritance of TNF and IL-6 gene polymorphisms [47–49]. TP53 is a tumor suppressor that is crucial for controlling the cell cycle and apoptosis. If TP53 is damaged, cancerous cell proliferation may result from aberrant cell proliferation. According to studies, the presence of TP53 overexpression in OLP indicates the presence of a setting that is favorable to malignant transformation and aids in determining the malignant potential of OLP [50, 51]. JUN is a member of the AP-1 transcription factor complex and has a significant impact on the growth of OLP [52]. Studies have shown that in lichen planus (LP), the activation level of c-Jun is between that of SCC and normal skin, suggesting that the activation of c-Jun is related to the malignant transformation, and the modulation and/or deregulation of apoptosis in the basal nucleus is thought to be mediated by c-Jun [53].

The etiology and pathogenesis of OLP are complex, and its development involves multiple biological processes and pathways. We used the R language to carry out GO and KEGG analysis to understand the mechanism of *Cordyceps sinensis* for OLP treatment. The biological processes involved in the shared targets mainly include regulation of apoptotic signaling pathway, T cell activation, epithelial cell proliferation, response to oxidative stress, regulation of lymphocyte activation, and others. It was hypothesized that the ability of *Cordyceps sinensis* for the treatment of OLP may be associated with the control of the body's immune system as well as cell growth and apoptosis, which coincides with the possible etiology and pathogenesis of OLP. Numerous investigations have demonstrated a connection between the pathophysiology of OLP and aberrant T cell activation, oral keratin-forming cell death, and the body's redox state [9, 11]. Additionally, KEGG pathway analysis showed that the two most prevalent signaling pathways were the PI3K-Akt signaling pathway (hsa04151) and the MAPK signaling pathway (hsa04010). Studies have shown that OLP-derived exosomes can regulate the OLP process through the PI3K/Akt signaling pathway [54] and that an aberrant PI3K/Akt signaling pathway might affect the interaction of T cells with keratinocytes and the cytokine network imbalance, contributing to the immunomodulatory mechanism of OLP [55]. Additionally, the carcinogenic potential of OLP is tightly connected to the PI3K/Akt signaling pathway [56]. Mitogen-activated protein kinase (MAPK) is one of the signaling pathways affected in cancers that regulates cell proliferation, differentiation, survival, and apoptosis [57]. OLP and OSCC tissue specimens had considerably greater levels of MAPK/ERK1/2 gene expression than healthy control specimens. In untreated precancerous lesions, higher levels of extracellular stimuli such as mitogens, inflammatory cytokines, and growth factors may raise the expression of the MAPK/ERK1/2 genes, increasing the likelihood of malignant transformation [58].

Molecular docking results showed that, except for arachidonic acid, the minimum binding energy between adenosine, beta-sitosterol, berberine, cordycepin, and their corresponding key targets AKT1, TNF, TP53, JUN, and IL-6 was less than −5 kcal/mol, indicating that there was a high affinity between the small molecule drugs and the target proteins. Therefore, these components could be the key pharmacological substances for *Cordyceps sinensis* to be effective. Previous research has demonstrated that the pathophysiology of OLP is related to a Th1/Th2 imbalance and berberine can suppress the imbalance between Th1 and Th2 cells [59, 60]. Additionally, following berberine therapy, the production of numerous anti-inflammatory cytokines, such as IL-10, was increased whereas other inflammatory cytokines, such as IL-1 and IL-6, were downregulated [61]. Furthermore, it was established that OS plays a role in the etiology of OLP, and patients with OLP had increased levels of salivary ROS, lipid peroxidation, nitric oxide, and nitrite [11]. To lessen the generation of reactive oxygen species, berberine demonstrated hydroxyl radical cleansing activity and ferrous ion chelating activity *in vitro* [62]. An isolated form of adenosine called cordycepin has been utilized as a medicinal supplement and medicine substitute. Cordycepin exerts its therapeutic effects mainly through activation of AMPK, inhibition of PI3K/mTOR/AKT, and suppression of inflammatory responses, which has excellent potential for OLP therapy [63]. *β*-sitosterol, also known as “the secret to life” is a phytosterol that is widely present in natural plants [64]. *β*-sitosterol has anti-inflammatory and antioxidant properties that can lower TNF and IL-1 levels and boost the activity of antioxidant enzymes such as catalase (CAT) and glutathione (GSH) [64]. These results supported the function and mode of action of active components of Cordyceps in the management of OLP.

In conclusion, adenosine, beta-sitosterol, berberine, and cordycepin are the key active substances of *Cordyceps sinensis* for the treatment of OLP. These components improve OLP by interfering with a number of targets (such as AKT1, TNF, TP53, JUN, and IL-6), biological processes (including apoptosis signaling pathway regulation, T cell activation, oxidative stress response), and signaling pathways (such as the PI3K-Akt signaling pathway and MAPK signaling pathway). More in vivo and in vitro testing are needed to confirm and investigate the efficacy of these active components, targets, and associated pathways identified by network pharmacology. Despite some limitations, this study provides good ideas and directions for future experimental validation and clinical treatment of OLP.

## Figures and Tables

**Table 1:** The active ingredients of *Cordyceps sinensis*.

No.	Molecule name	OB (%)	DL	No	Molecule name	OB (%)	DL
C1	Arachidonic acid	45.57	0.20	C14	Isotalatizidine	50.82	0.73
C2	Linoleyl acetate	42.10	0.20	C15	Neokadsuranic acid C	35.40	0.85
C3	Beta-sitosterol	36.91	0.75	C16	Karakoline	51.73	0.73
C4	Peroxyergosterol	44.39	0.82	C17	Vilmorrianine C	33.96	0.22
C5	Cerevisterol	39.52	0.77	C18	Styrone	38.35	0.22
C6	Cholesteryl palmitate	31.05	0.45	C19	Deltoin	46.69	0.37
C7	CLR	37.87	0.68	C20	Karanjin	69.56	0.34
C8	Hypaconitine	31.39	0.26	C21	Crassicauline A	34.13	0.21
C9	Berberine	36.86	0.78	C22	MTL	17.73	0.03
C10	Deoxyaconitine	30.96	0.24	C23	Adenosine	15.98	0.18
C11	Ignavine	84.08	0.25	C24	Ergosterol	14.29	0.72
C12	3-Acetylaconitine	37.05	0.20	C25	Inosine	11.17	0.18
C13	Deoxyandrographolide	56.30	0.31	C26	Cordycepin	38.44	0.16

**Table 2:** Topological parameters of key target sites.

Target	Degree	Closeness	Betweenness	Target	Degree	Closeness	Betweenness
AKT1	38	51	473	CASP3	26	45	102
TNF	36	51	418	HRAS	24	44	105
TP53	34	50	335	IGF1	24	44	99
JUN	33	49	398	INS	23	44	152
IL-6	32	49	149	RELA	22	43	103
VEGFA	30	48	138	ESR1	21	42	110
EGFR	30	48	196	TGFB1	20	42	275
IL1B	28	47	108	IL4	18	41	190
EGF	26	45	95	PTGS2	15	40	123
ALB	26	45	233				

**Table 3:** Docking parameters and results.

No.	Target	PDB ID	Compound	Minimum binding energy (kcal/mol)
*a*	AKT1	1h10	Adenosine	−6.10
*b*	AKT1	1h10	Arachidonic acid	−4.30
*c*	TNF	5uui	Berberine	−6.00
*d*	TP53	1yc5	Berberine	−6.20
*e*	JUN	6y3v	Beta-sitosterol	−6.10
*f*	JUN	6y3v	Cordycepin	−5.30
*g*	JUN	6y3v	Berberine	−5.30
*h*	IL-6	4ni7	Berberine	−6.80

## Data Availability

The data used to support the findings of this study are available from the corresponding author upon request.

## Supplementary Materials

Supplementary Materials Supplementary table 1: The summary of putative targets of *Cordyceps sinensis*. Supplementary table 2: The 293 OLP-related human genes. Supplementary table 3: The topological parameter of 52 significant OLP-related targets. Supplementary table 4: The 67 common targets of *Cordyceps sinensis* and OLP. Supplementary table 5: The top 10 biological processes, cellular components, and molecular function. Supplementary table 6: The top 20 signaling pathways.
