# Supplementary material for: Network Pharmacology and Molecular Docking Analysis Explores the Mechanisms of Cordyceps sinensis in the Treatment of Oral Lichen Planus
Source: J Oncol. 2022 Aug 29;2022:3156785. doi: 10.1155/2022/3156785 (PMC9444403; doi:10.1155/2022/3156785)
Supplement: Supplementary Materials — Supplementary table 1: The summary of putative targets of Cordyceps sinensis. Supplementary table 2: The 293 OLP-related human genes. Supplementary table 3: The topological parameter of 52 significant OLP-related targets. Supplementary table 4: The 67 common targets of Cordyceps sinensis and OLP. Supplementary table 5: The top 10 biological processes, cellular components, and molecular function. Supplementary table 6: The top 20 signaling pathways. [file 3156785.f1.zip › Table 1 (1).pdf]

Supplement 1. The summary of putative targets of Cordyceps sinensis
LSS
CYP2C9
RARB
HNF4G
OAT
FABP7
CYP2C8
FOLH1
TRAPPC3
FABP3
GRB2
PROCR
HSD11B1
MET
ADK
F10
ADH5
SEC14L2
RORA
HSP90AB1
ABO
PYGL
ADAM17
BCL2L1
MMP2
RBP4
ERBB4
DPP4
CRABP2
RXRB
ITGAL
ITK
RARG
FABP4
MDM2
S100A9
BLVRB
PLA2G2A
TYMS
NR1H3
PDE5A
JAK3
NR3C2
ESRRA
VDR
PTPN11
SERPINA1
GM2A
SULT2B1
GC

NR3C1
FABP5
HDAC8
PPARA
ESRRG
CDK2
ANXA5
MAPK14
KIF11
TPSB2
GSTA1
NR1I3
PCTP
FECH
PDPK1
THRA
NR1H4
WAS
TTPA
MAP2K1
HMOX1
HSD17B1
BHMT
HNMT
SULT2A1
SYK
KDR
FABP6
PGR
CASP3
F2
ABL1
FGFR1
PIK3R1
RXRA
FNTA
GSTP1
SHBG
IL2
AKR1C1
PARP1
REN
PDE4D
EPHB4
HSP90AA1
NR1H2
PDE3B
NR1I2
RARA
GSK3B
MMP13

PPARG
CA2
ADH1C
PDK2
PPP1CC
XIAP
MAOA
MMP8
CTSK
ELANE
FKBP1A
MMP3
PPARD
HMGCR
ALB
CHEK1
DCK
TTR
CFD
PDE4B
PTPN1
NQO1
AKR1B1
AR
CCNA2
PADI4
LTA4H
MMP12
BACE1
CHIT1
PIM1
LCK
F11
ACADM
FGFR2
CASP1
MTHFD1
EGFR
JAK2
ESR2
MAOB
PAH
SOD2
ESR1
SORD
PCK1
MTAP
GSTA3
LCN2
DHODH
AKR1C3

THRB
MME
FKBP1B
NOS3
MAPK1
MAPK8
YARS
ALAD
PLA2G10
CD1A
KIT
DPEP1
AMD1
MAPK10
INSR
SRC
CTSB
AURKA
TGFBR1
BIRC7
CTNNA1
CES1
FKBP3
TEK
F7
AMY2A
PIK3CG
CTSF
IGF1R
PSAP
CTSS
CA1
SHMT1
TGFB2
HCK
PNMT
DHFR
MAPKAPK2
DTYMK
GSTM1
KAT2B
GART
AKR1C2
GCK
BST1
AMY1B
EPHX2
PGF
BCAT2
RAN
PLK1

TGM3
PRKCQ
FDPS
SULT1E1
ZAP70
AMY1A
GLO1
TRDMT1
NQO2
CMA1
PPIA
MIF
HSPA8
BTK
EIF4E
CBR1
NT5M
CTSG
CDK6
MMP9
IGLV2-8
AMY1B
MAN1B1
G6PD
GALK1
PLAU
IMPA1
HK1
GNPDA1
GP1BA
NOS2
PNP
RAB5A
RAB11A
UAP1
CLEC4M
ANG
GMPR2
LGALS2
IMPDH2
HEXB
AMY1C
RAC1
CD209
HRAS
GMPR
ACPP
UCK2
HAGH
SRM
LGALS3

HINT1
GPI
ARG2
AHCY
RAP2A
STAT1
RAB9A
APRT
RNASE3
GBA
ITPKA
AK1
EPHA2
DOT1L
DCPS
PKLR
WARS
AKT1
APAF1
CDK5R1
TGFBR2
MMP7
FGG
ADAM33
HPRT1
TPH1
CDA
HADH
DAPK1
MMP1
TNK2
ATOX1
CLK1
SULT1A1
ERI1
MMP16
TAP1
RHOA
GALE
RELA
ABCG1
ABCC4
HERC5
CDK4
KCNK10
GLB1
PTGS2
PLA2G4A
SELP
TNFRSF1A
TNFRSF1B

PTGES
ALDH3A1
PTGES2
PTEN
PPP5C
RETN
ALOX5
PECAM1
ABCA1
KCNK2
ALDH2
UCP2
CETP
COL1A2
C1R
PRKCB
EGF
APOD
CYP1B1
F2R
TRPC4
SOD1
TRPM8
SMO
MAPK3
EGR1
YWHAZ
NFE2L2
PLA2G1B
XDH
PTGS1
SCNN1A
ASIC3
TRPV4
IDO1
SH3GL2
VIM
TWIST1
MAP2
BCL2
CASP9
BAX
JUN
PON1
CASP8
PRKCA
TGFB1
PRKAA2
EDN1
DHCR7
TNF

CCL2
IL6
CXCL12
ABCG2
GAL
HNF4A
INS
RASGRF2
CDC42
TP53
CDKN1A
RUNX1T1
EIF6
PCSK9
BID
ALDH18A1
DDIT3
TEP1
SLC2A1
APP
IL1B
AGT
FOS
PDGFA
CCNB1
WEE1
CDK1
CYCS
NFKBIA
CYP1A1
AHR
CD69
IL2RA
VEGFA
HIF1A
IL4
IFNG
MGAM
SI
CXCL8
MPO
TH
ACHE
PDK4
GATA2
ADORA3
P2RY1
NT5E
