# Supplementary material for: Network Pharmacology and Molecular Docking Analysis Explores the Mechanisms of Cordyceps sinensis in the Treatment of Oral Lichen Planus
Source: J Oncol. 2022 Aug 29;2022:3156785. doi: 10.1155/2022/3156785 (PMC9444403; doi:10.1155/2022/3156785)
Supplement: Supplementary Materials — Supplementary table 1: The summary of putative targets of Cordyceps sinensis. Supplementary table 2: The 293 OLP-related human genes. Supplementary table 3: The topological parameter of 52 significant OLP-related targets. Supplementary table 4: The 67 common targets of Cordyceps sinensis and OLP. Supplementary table 5: The top 10 biological processes, cellular components, and molecular function. Supplementary table 6: The top 20 signaling pathways. [file 3156785.f1.zip › Table 2 (1).pdf]

Supplement 2. The 293 OLP-related human genes.

IL10	DisGeNET,GeneCard
TP53	DisGeNET,GeneCard
TNF	DisGeNET,GeneCard
IFNG	DisGeNET,GeneCard
TLR4	DisGeNET,GeneCard
IL6	DisGeNET,GeneCard
TLR2	DisGeNET,GeneCard
MMP9	DisGeNET,GeneCard
CXCL8	DisGeNET,GeneCard
IL4	DisGeNET,GeneCard
PTGS2	DisGeNET,GeneCard
FOXP3	DisGeNET,GeneCard
MIR27B	DisGeNET,GeneCard
IL1B	DisGeNET,GeneCard
TP63	DisGeNET,GeneCard
IL17A	DisGeNET,GeneCard
MIR155	DisGeNET,GeneCard
MIR203A	DisGeNET,GeneCard
RPE65	DisGeNET
UVRAG	DisGeNET
BCL2	DisGeNET,GeneCard
CKAP4	DisGeNET
CCL5	DisGeNET,GeneCard
MTCO2P1	DisGeNET
IGF1	DisGeNET,GeneCard
H3P10	DisGeNET
CDKN2A	DisGeNET,GeneCard
VDR	DisGeNET,GeneCard
CCR5	DisGeNET,GeneCard
MTOR	DisGeNET,GeneCard
CD44	DisGeNET,GeneCard
HMGB1	DisGeNET,GeneCard
HIF1A	DisGeNET,GeneCard
IL22	DisGeNET
CYP2D6	DisGeNET,GeneCard
MIR137	DisGeNET,GeneCard
COX2	DisGeNET
DEFB4A	DisGeNET,GeneCard
MIR21	DisGeNET,GeneCard
CXCL10	DisGeNET,GeneCard
MIR146A	DisGeNET,GeneCard
SPP1	DisGeNET,GeneCard
TLR3	DisGeNET
KRT19	DisGeNET,GeneCard
MIR125A	DisGeNET
IL18	DisGeNET,GeneCard
TERC	DisGeNET
MBL2	DisGeNET,GeneCard
TGFB1	DisGeNET,GeneCard
IL9	DisGeNET

CXCL9	DisGeNET,GeneCard
ACTB	DisGeNET
ISG20	DisGeNET
KDR	DisGeNET
MDM2	DisGeNET,GeneCard
TG	DisGeNET,GeneCard
IL2RA	DisGeNET,GeneCard
CXCR3	DisGeNET,GeneCard
TSLP	DisGeNET
MAP1LC3	DisGeNET
CDKN1A	DisGeNET,GeneCard
PPIG	DisGeNET
AKT1	DisGeNET,GeneCard
NLRP3	DisGeNET
IL23A	DisGeNET
VEGFA	DisGeNET,GeneCard
CD14	DisGeNET,GeneCard
DEFB4B	DisGeNET
IL33	DisGeNET,GeneCard
CRP	DisGeNET
COL17A1	GeneCard
DSG3	GeneCard
KRT13	GeneCard
ICAM1	GeneCard
OSMR	GeneCard
MMP1	GeneCard
IL2	GeneCard
EGFR	GeneCard
CASP3	GeneCard
KRT16	GeneCard
IL1A	GeneCard
HRAS	GeneCard
RET	GeneCard
MMP2	GeneCard
KRT4	GeneCard
INS	GeneCard
LAMC2	GeneCard
DST	GeneCard
COL7A1	GeneCard
VCAM1	GeneCard
BAX	GeneCard
CPLANE1	GeneCard
PIK3CA	GeneCard
KRT14	GeneCard
KRT1	GeneCard
HSPA4	GeneCard
GZMB	GeneCard
HLA-B	GeneCard
CDH1	GeneCard
HLA-DRA	GeneCard
FLG	GeneCard

TGFBR2	GeneCard
HSPD1	GeneCard
TIMP2	GeneCard
ITGB4	GeneCard
KRT10	GeneCard
PCNA	GeneCard
HSPA8	GeneCard
TERT	GeneCard
CD1A	GeneCard
ALB	GeneCard
GPNMB	GeneCard
RB1	GeneCard
IVL	GeneCard
TGFBR1	GeneCard
FAS	GeneCard
FBN1	GeneCard
HLA-DQE	GeneCard
CD58	GeneCard
DSG1	GeneCard
IFNA1	GeneCard
ITGAL	GeneCard
HLA-DRE	GeneCard
KRT5	GeneCard
CAT	GeneCard
MX1	GeneCard
IRF6	GeneCard
NTRK1	GeneCard
HLA-A	GeneCard
HMGCR	GeneCard
MMP3	GeneCard
BRCA1	GeneCard
ABCB1	GeneCard
CCL2	GeneCard
CYP2C9	GeneCard
PTEN	GeneCard
ELN	GeneCard
TGM1	GeneCard
FGFR1	GeneCard
CDKN1B	GeneCard
TGFA	GeneCard
CYP2C19	GeneCard
CD8A	GeneCard
CD4	GeneCard
CD36	GeneCard
SHBG	GeneCard
DEFB1	GeneCard
DSC1	GeneCard
MKI67	GeneCard
MTHFR	GeneCard
F13A1	GeneCard
MPO	GeneCard

PDPN	GeneCard
SERPINC	GeneCard
LAMB3	GeneCard
IL12A	GeneCard
DKC1	GeneCard
IFNB1	GeneCard
CD40LG	GeneCard
CCR1	GeneCard
PPARG	GeneCard
IL5	GeneCard
CDK4	GeneCard
IL1RN	GeneCard
CCR6	GeneCard
DEFB103]	GeneCard
ECM1	GeneCard
SELE	GeneCard
ESR1	GeneCard
SOD1	GeneCard
CTNNB1	GeneCard
AIRE	GeneCard
ABCG2	GeneCard
FASLG	GeneCard
MYC	GeneCard
H2AC18	GeneCard
TGIF1	GeneCard
EGF	GeneCard
GSTP1	GeneCard
SUMO1	GeneCard
ERBB2	GeneCard
FGFR3	GeneCard
CYP1A2	GeneCard
SLC6A4	GeneCard
CASP8	GeneCard
BMP4	GeneCard
GPT	GeneCard
MGMT	GeneCard
GCG	GeneCard
SLC17A5	GeneCard
RELA	GeneCard
CALCA	GeneCard
CSF2	GeneCard
INSR	GeneCard
NFKB1	GeneCard
RARB	GeneCard
XYLT1	GeneCard
BIRC5	GeneCard
JUN	GeneCard
GGT1	GeneCard
ADH1C	GeneCard
LEP	GeneCard
CD79A	GeneCard

ACVRL1	GeneCard
JAK3	GeneCard
CSF3	GeneCard
CD40	GeneCard
NGF	GeneCard
CD274	GeneCard
TLR9	GeneCard
SELL	GeneCard
KRAS	GeneCard
TRPV1	GeneCard
ABCC6	GeneCard
AHR	GeneCard
RIPK1	GeneCard
IGFBP3	GeneCard
F3	GeneCard
CD34	GeneCard
GH1	GeneCard
TAC1	GeneCard
DPP4	GeneCard
CYP21A2	GeneCard
SOX2	GeneCard
AR	GeneCard
MIR29A	GeneCard
SERPINB:	GeneCard
DSP	GeneCard
FOS	GeneCard
XYLT2	GeneCard
ACTC1	GeneCard
CPT2	GeneCard
IL31	GeneCard
PRF1	GeneCard
LTA	GeneCard
TTR	GeneCard
CD27	GeneCard
MYLK	GeneCard
MIR29B1	GeneCard
RYR1	GeneCard
MMP14	GeneCard
HLA-C	GeneCard
POMC	GeneCard
IFNA2	GeneCard
BCL2L1	GeneCard
CA9	GeneCard
SELP	GeneCard
FGF2	GeneCard
TNFRSF1	GeneCard
ITGA2	GeneCard
DEFA1	GeneCard
MIR27A	GeneCard
ADIPOQ	GeneCard
KRT2	GeneCard

PDCD1	GeneCard
PECAM1	GeneCard
CD69	GeneCard
IL2RB	GeneCard
MYD88	GeneCard
DNMT3B	GeneCard
MIR122	GeneCard
LORICRI	GeneCard
CD1E	GeneCard
CCK	GeneCard
TTN	GeneCard
MIR19A	GeneCard
TACR1	GeneCard
FZD6	GeneCard
GNLY	GeneCard
MIR26B	GeneCard
TP73	GeneCard
ERCC1	GeneCard
PDGFRB	GeneCard
CCR4	GeneCard
CAV1	GeneCard
ALDH1A	GeneCard
ITGB2	GeneCard
SYP	GeneCard
IRS1	GeneCard
FCGR3B	GeneCard
CCR7	GeneCard
MEN1	GeneCard
ITGA3	GeneCard
NGFR	GeneCard
SMAD7	GeneCard
MIF	GeneCard
CD38	GeneCard
IGFBP1	GeneCard
FIP1L1	GeneCard
KRT15	GeneCard
DMD	GeneCard
C3	GeneCard
CXCL1	GeneCard
