# Supplementary material for: Network Pharmacology and Molecular Docking Analysis Explores the Mechanisms of Cordyceps sinensis in the Treatment of Oral Lichen Planus
Source: J Oncol. 2022 Aug 29;2022:3156785. doi: 10.1155/2022/3156785 (PMC9444403; doi:10.1155/2022/3156785)
Supplement: Supplementary Materials — Supplementary table 1: The summary of putative targets of Cordyceps sinensis. Supplementary table 2: The 293 OLP-related human genes. Supplementary table 3: The topological parameter of 52 significant OLP-related targets. Supplementary table 4: The 67 common targets of Cordyceps sinensis and OLP. Supplementary table 5: The top 10 biological processes, cellular components, and molecular function. Supplementary table 6: The top 20 signaling pathways. [file 3156785.f1.zip › Table 3 (1).pdf]

:ameter of 52 significant OLP-related targets.

Gene	Degree	Betweenness Centrality	Closeness Centrality
TNF	97	3961.27947	174.58333
IL6	86	2917.72259	169.25
CD4	75	3363.9721	160.83333
EGFR	73	5126.4444	163.33333
IL1B	72	1412.23644	160.66667
IL10	70	1412.55088	159.25
AKT1	69	2694.5633	160.75
VEGFA	66	1572.88131	157.66667
TP53	64	3298.83181	156.66667
IL2	65	1705.2	155.25
ACTB	61	2666.82879	156.75
CD8A	61	1877.80198	150.33333
ALB	60	3303.27204	155.41667
IL4	57	1463.29923	150.91667
INS	58	2708.62162	152.66667
JUN	57	2139.07331	154
CXCL8	57	786.6826	149.75
CTNNB1	56	2930.86645	150.33333
PIK3CA	56	1737.10795	152.16667
IFNG	56	641.07604	148.75
CD44	55	3126.60237	153.41667
CCL2	53	874.69482	146
TLR4	50	2107.18692	149.08333
RELA	52	1262.92787	149.16667
CASP3	50	2492.63444	149.08333
HRAS	49	563.76981	147.16667
IL17A	49	432.03669	142
EGF	48	617.86875	147.91667
IGF1	47	941.78194	145.66667
MMP9	45	1041.04582	145.58333
CCL5	45	447.80915	139.75
MYC	43	525.88133	142.91667
FGF2	42	468.85832	143.08333
ESR1	41	1160.11543	140.66667
CAV1	38	2013.49848	143.16667
ICAM1	40	491.39752	140.25
TLR2	39	984.68523	141.25
ERBB2	39	795.21876	143.16667
HIF1A	39	470.85878	140.5
CDH1	38	1062.98789	141.58333
TGFB1	33	834.2747	138.5
MYD88	31	907.26322	131.58333
MMP2	30	660.39559	134.66667
FOS	30	611.94498	134.91667
MTOR	30	634.53885	133.66667
CRP	29	441.47369	128.75
AR	27	698.0675	130.66667
NGF	28	749.83033	132.75
PTGS2	25	1140.17548	135.83333

CASP8	23	484.13075	128.75
PDGFRB	21	556.12025	128.5
POMC	21	770.64767	123.61667
