# Supplementary material for: Network Pharmacology and Molecular Docking Analysis Explores the Mechanisms of Cordyceps sinensis in the Treatment of Oral Lichen Planus
Source: J Oncol. 2022 Aug 29;2022:3156785. doi: 10.1155/2022/3156785 (PMC9444403; doi:10.1155/2022/3156785)
Supplement: Supplementary Materials — Supplementary table 1: The summary of putative targets of Cordyceps sinensis. Supplementary table 2: The 293 OLP-related human genes. Supplementary table 3: The topological parameter of 52 significant OLP-related targets. Supplementary table 4: The 67 common targets of Cordyceps sinensis and OLP. Supplementary table 5: The top 10 biological processes, cellular components, and molecular function. Supplementary table 6: The top 20 signaling pathways. [file 3156785.f1.zip › Table 4 (1).pdf]

Supplement 4. The 67 common targets of Cordyceps sinensis and OLP

Gene symbol

CYP2C9
RARB
IGF1
BCL2L1
MMP2
DPP4
ITGAL
MDM2
JAK3
VDR
KDR
CASP3
FGFR1
GSTP1
SHBG
IL2
PPARG
ADH1C
MMP3
ALB
TTR
AR
EGFR
ESR1
CD1A
INSR
TGFBR1
MIF
HSPA8
MMP9
HRAS
AKT1
TGFBR2
MMP1
RELA
CDK4
PTGS2
SELP
TNFRSF1B
PTEN
PECAM1
EGF
SOD1
BCL2
BAX
JUN
CASP8
TGFB1
TNF

CCL2
IL6
ABCG2
INS
TP53
CDKN1A
IL1B
FOS
AHR
CD69
IL2RA
VEGFA
HIF1A
IL4
IFNG
CXCL8
MPO
CCND1
