# Supplementary material for: Network Pharmacology and Molecular Docking Analysis Explores the Mechanisms of Cordyceps sinensis in the Treatment of Oral Lichen Planus
Source: J Oncol. 2022 Aug 29;2022:3156785. doi: 10.1155/2022/3156785 (PMC9444403; doi:10.1155/2022/3156785)
Supplement: Supplementary Materials — Supplementary table 1: The summary of putative targets of Cordyceps sinensis. Supplementary table 2: The 293 OLP-related human genes. Supplementary table 3: The topological parameter of 52 significant OLP-related targets. Supplementary table 4: The 67 common targets of Cordyceps sinensis and OLP. Supplementary table 5: The top 10 biological processes, cellular components, and molecular function. Supplementary table 6: The top 20 signaling pathways. [file 3156785.f1.zip › Table 5 (1).pdf]

Supplement 5. The top 10 of Biological proccess,Cellular component and Molecular function

GOterm	subgroup	Gene number
extrinsic apoptotic signaling pathway	Biological process	19
regulation of apoptotic signaling pathway	Biological process	22
gland development	Biological process	22
negative regulation of apoptotic signaling pathway	Biological process	18
regulation of reactive oxygen species metabolic process	Biological process	17
muscle cell proliferation	Biological process	18
T cell activation	Biological process	22
regulation of smooth muscle cell proliferation	Biological process	16
smooth muscle cell proliferation	Biological process	16
response to lipopolysaccharide	Biological process	19
membrane raft	Cellular component	13
membrane microdomain	Cellular component	13
membrane region	Cellular component	13
vesicle lumen	Cellular component	12
secretory granule lumen	Cellular component	11
cytoplasmic vesicle lumen	Cellular component	11
platelet alpha granule	Cellular component	7
transcription factor complex	Cellular component	10
RNA polymerase II transcription factor complex	Cellular component	7
platelet alpha granule lumen	Cellular component	5
cytokine receptor binding	Molecular function	15
receptor ligand activity	Molecular function	15
cytokine activity	Molecular function	11
ubiquitin protein ligase binding	Molecular function	11
ubiquitin-like protein ligase binding	Molecular function	11
nuclear receptor activity	Molecular function	6
transcription factor activity	Molecular function	6
protease binding	Molecular function	8
transmembrane receptor protein kinase activity	Molecular function	6
growth factor receptor binding	Molecular function	7
