# Supplementary material for: Network Pharmacology and Molecular Docking Analysis Explores the Mechanisms of Cordyceps sinensis in the Treatment of Oral Lichen Planus
Source: J Oncol. 2022 Aug 29;2022:3156785. doi: 10.1155/2022/3156785 (PMC9444403; doi:10.1155/2022/3156785)
Supplement: Supplementary Materials — Supplementary table 1: The summary of putative targets of Cordyceps sinensis. Supplementary table 2: The 293 OLP-related human genes. Supplementary table 3: The topological parameter of 52 significant OLP-related targets. Supplementary table 4: The 67 common targets of Cordyceps sinensis and OLP. Supplementary table 5: The top 10 biological processes, cellular components, and molecular function. Supplementary table 6: The top 20 signaling pathways. [file 3156785.f1.zip › Table 6 (1).pdf]

Supplement 6. The top 20 of signaling pathways

ID	Description	GeneRatio	pvalue	Count
hsa04151	PI3K-Akt signaling pathway	24/65	9.02826E-17	24
hsa05166	Human T-cell leukemia virus 1 infection	21/65	1.01961E-17	21
hsa04010	MAPK signaling pathway	21/65	4.28665E-15	21
hsa04933	AGE-RAGE signaling pathway in diabetic complications	19/65	7.54756E-22	19
hsa05161	Hepatitis B	19/65	1.0634E-17	19
hsa05163	Human cytomegalovirus infection	19/65	5.28779E-15	19
hsa05215	Prostate cancer	18/65	1.64685E-20	18
hsa05162	Measles	18/65	1.43828E-17	18
hsa05205	Proteoglycans in cancer	18/65	1.58304E-14	18
hsa05167	Kaposi sarcoma-associated herpesvirus infection	17/65	9.13455E-14	17
hsa05165	Human papillomavirus infection	17/65	5.37681E-10	17
hsa04657	IL-17 signaling pathway	16/65	1.23077E-17	16
hsa01522	Endocrine resistance	16/65	2.48374E-17	16
hsa05418	Fluid shear stress and atherosclerosis	16/65	7.81827E-15	16
hsa05169	Epstein-Barr virus infection	16/65	2.85742E-12	16
hsa05210	Colorectal cancer	15/65	9.74267E-17	15
hsa05142	Chagas disease	15/65	1.42011E-15	15
hsa04659	Th17 cell differentiation	15/65	2.98096E-15	15
hsa04068	FoxO signaling pathway	15/65	6.55291E-14	15
hsa05224	Breast cancer	15/65	3.68883E-13	15
